# Supplementary material for: Assessing Physical Activity and Sedentary Behavior under Free-Living Conditions: Comparison of Active Style Pro HJA-350IT and ActiGraphTM GT3X+
Source: Int J Environ Res Public Health. 2019 Aug 23;16(17):3065. doi: 10.3390/ijerph16173065 (PMC6747387; doi:10.3390/ijerph16173065)
Supplement: Supplementary file 1 [file ijerph-16-03065-s001.pdf]

(a) Work day: Active style Pro and ActiGraph vertical axis

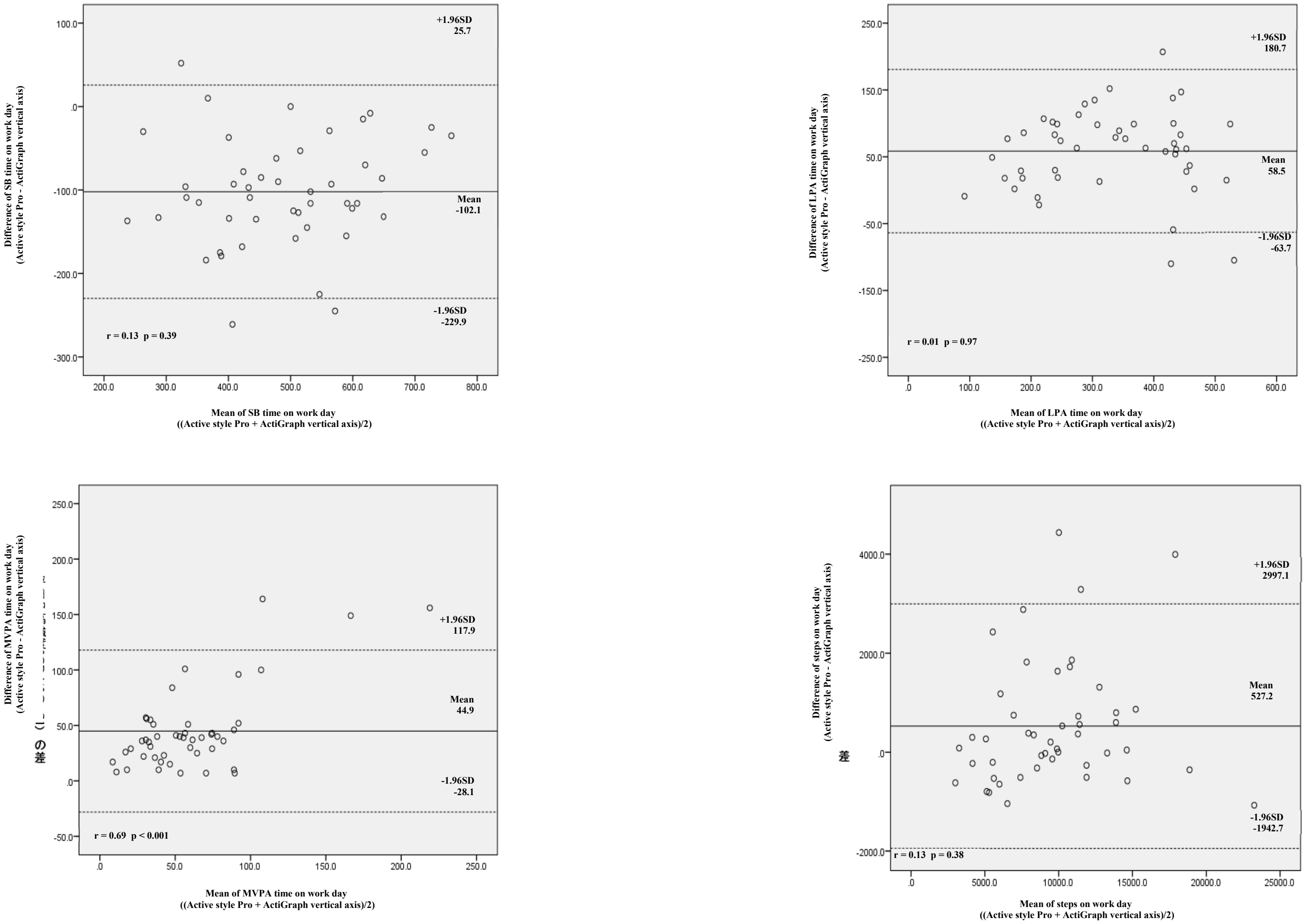

(b) Nonwork day: Active style Pro and ActiGraph vertical axis

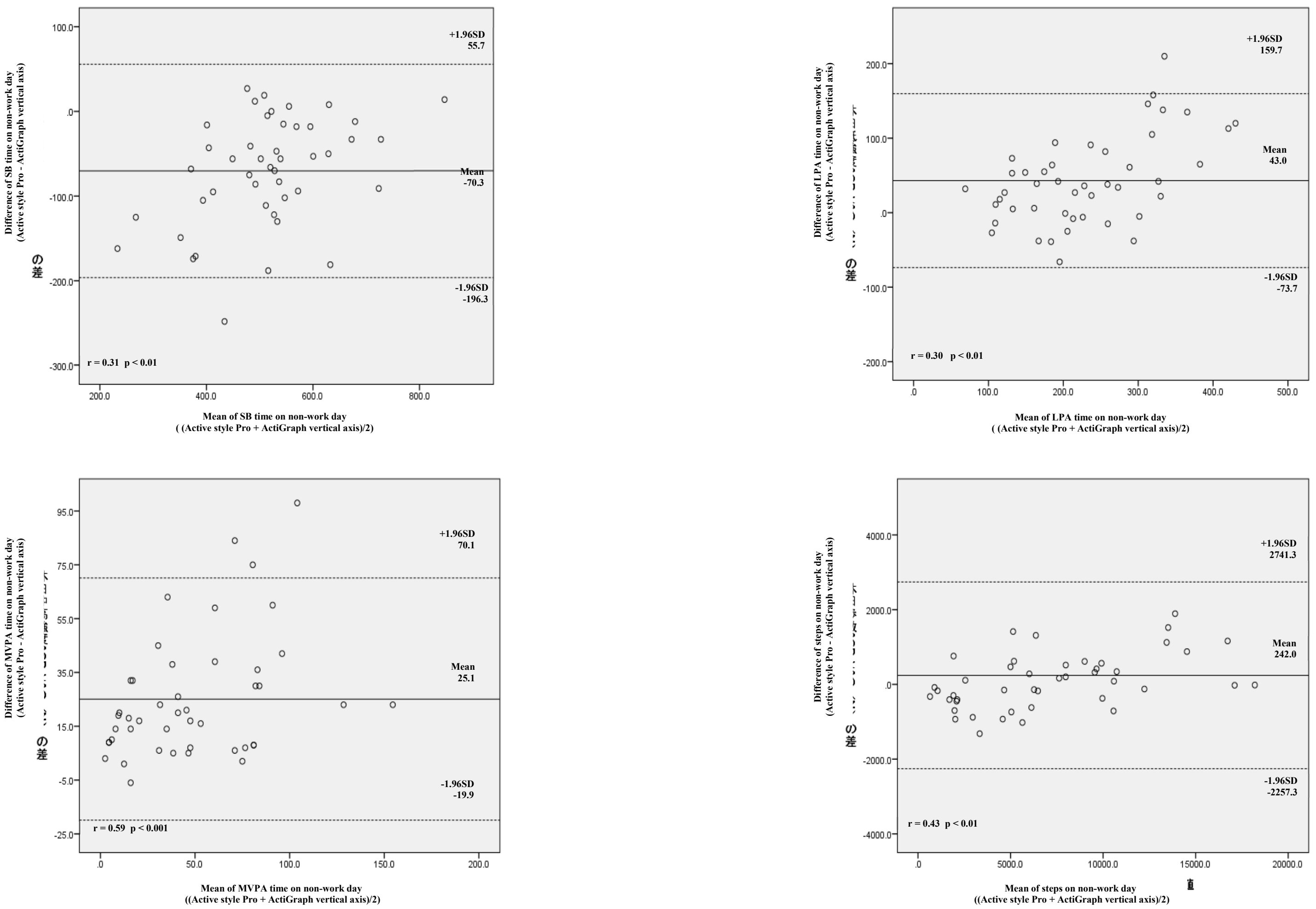

(c) Work day: Active style Pro and ActiGraph vector magnitude

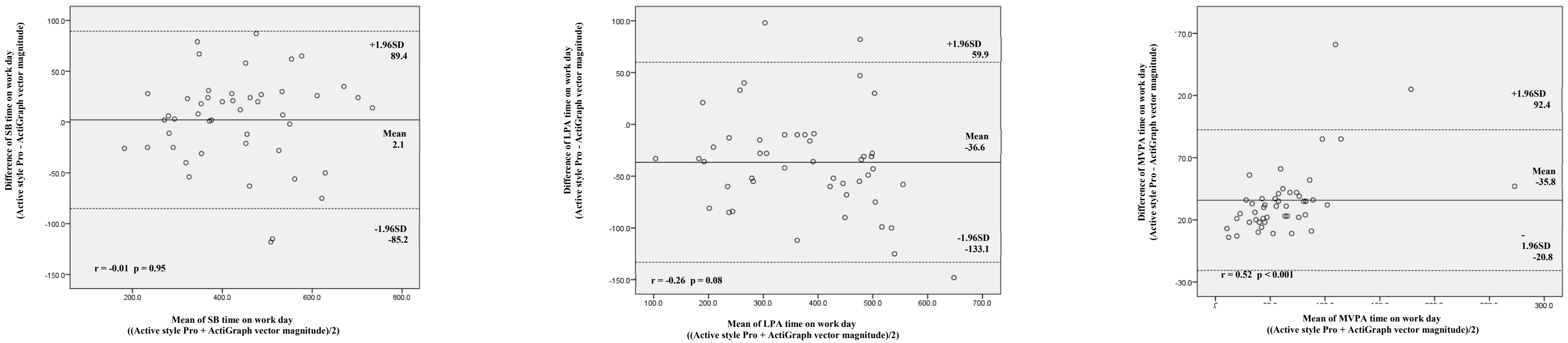

(d) Nonwork day: Active style Pro and ActiGraph vector magnitude

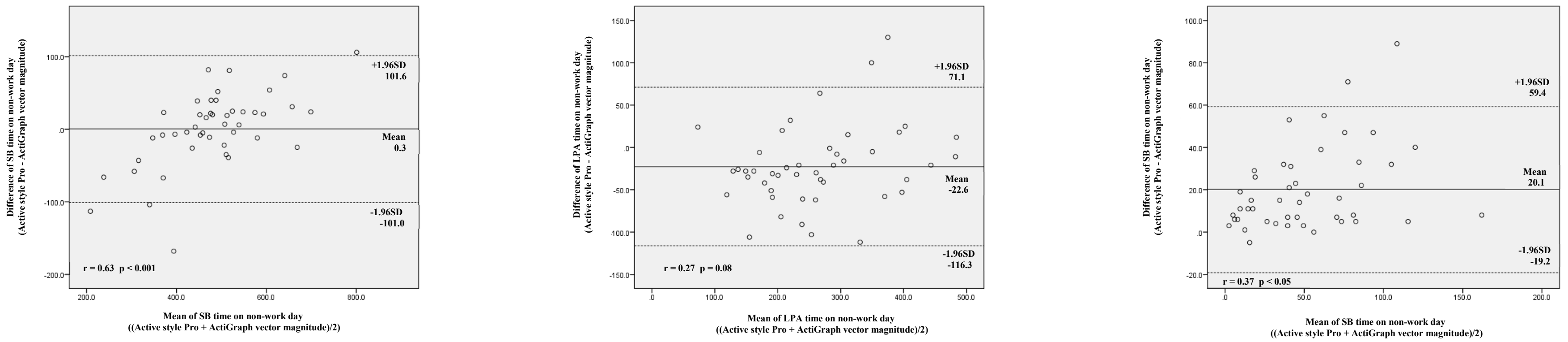

Figure S1. Bland Altman analysis for the agreement of data assessed with Active style Pro and ActiGraph: (a) Work day: Active style Pro and ActiGraph vertical axis, (b) Nonwork day: Active style Pro and ActiGraph vertical axis, (c) Work day: Active style Pro and ActiGraph vector magnitude, (d) Nonwork day: Active style Pro and ActiGraph vector magnitude.
